# Adverse pregnancy and perinatal outcomes associated with *Mycoplasma genitalium:* systematic review and meta-analysis

**DOI:** 10.1136/sextrans-2021-055352

**Published:** 2022-03-29

**Authors:** Carole Frenzer, Dianne Egli-Gany, Lisa M Vallely, Andrew J Vallely, Nicola Low

**Affiliations:** 1 Institute of Social and Preventive Medicine, University of Bern, Bern, Switzerland; 2 Kirby Institute for Infection and Immunity in Society, Faculty of Medicine, UNSW Sydney, Sydney, New South Wales, Australia; 3 Sexual and Reproductive Health Unit, Papua New Guinea Institute of Medical Research, Goroka, Eastern Highlands, Papua New Guinea

**Keywords:** mycoplasma, pregnancy, systematic review, meta-analysis

## Abstract

**Objective:**

To examine associations between *Mycoplasma genitalium* infection during pregnancy and adverse outcomes.

**Methods:**

We did a systematic review of observational studies. We searched Medline, EMBASE, the Cochrane Library and CINAHL up to 11 August 2021. Studies were included if they compared preterm birth, spontaneous abortion, premature rupture of membranes, low birth weight or perinatal death between women with and without *M. genitalium*. Two reviewers independently assessed articles for inclusion and extracted data. We used random-effects meta-analysis to estimate summary ORs and adjusted ORs, with 95% CIs, where appropriate. Risk of bias was assessed using established checklists.

**Results:**

We identified 116 records and included 10 studies. Women with *M. genitalium* were more likely to experience preterm birth in univariable analyses (summary unadjusted OR 1.91, 95% CI 1.29 to 2.81, I^2^=0%, 7 studies). The combined adjusted OR was 2.34 (95% CI 1.17 to 4.71, I^2^=0%, 2 studies). For spontaneous abortion, the summary unadjusted OR was 1.00 (95% CI 0.53 to 1.89, I^2^=0%, 6 studies). The adjusted OR in one case–control study was 0.9 (95% CI 0.2 to 3.8). Unadjusted ORs for premature rupture of membranes were 7.62 (95% CI 0.40 to 145.86, 1 study) and for low birth weight 1.07 (95% CI 0.02 to 10.39, 1 study). For perinatal death, the unadjusted OR was 1.07 (95% CI 0.49 to 2.36) in one case–control and 38.42 (95% CI 1.45 to 1021.43) in one cohort study. These two ORs were not combined, owing to heterogeneity. The greatest risk of bias was the failure in most studies to control for confounding.

**Conclusion:**

*M. genitalium* might be associated with an increased risk of preterm birth. Further prospective studies, with adequate control for confounding, are needed to understand the role of *M. genitalium* in adverse pregnancy outcomes. There is insufficient evidence to indicate routine testing and treatment of asymptomatic *M. genitalium* in pregnancy.

**PROSPERO registration number:**

CRD42016050962.

## Introduction

Bacterial STIs during pregnancy, such as *Chlamydia trachomatis* and *Neisseria gonorrhoeae* have been reported to be associated with one or more of the following adverse pregnancy and perinatal outcomes: spontaneous abortion, preterm birth (PTB), premature rupture of membranes (PROM), low birth weight (LBW) and perinatal death.^1–6^ In pregnancy, the inflammatory response resulting from infections that ascend to the upper genital tract provides a plausible biological mechanism for the association between STIs and preterm birth.^7^ It is hypothesised that preterm labour is a common pathway of a cascade of proinflammatory cytokine production, for which endocervical pathogens are one of the triggers.^7^ If associations observed in epidemiological studies reflect a causal pathway, early detection and treatment of STIs in pregnancy is a potential intervention. In observational epidemiological studies, it is essential to understand whether there are confounding factors that are known to be associated with both an exposure (eg, an STI) and an outcome (eg, preterm birth) and to control for them in multivariable statistical analyses. Systematic reviews show that potential confounders, such as young age, lower socioeconomic position and smoking are often not controlled for, however.^5 6^

*Mycoplasma genitalium* is the most recently identified bacterial STI. The prevalence of *M. genitalium* in high-income countries is around 1% in studies among the general population and is similar among pregnant women,^8^ but *M. genitalium* has been found in 12% or more of pregnant women in studies in South Africa and Papua New Guinea.^9 10^ The strength of association between *M. genitalium* during pregnancy and poor pregnancy outcomes is still unclear.^11^ In a systematic review of observational studies published up to 2014, Lis *et al* found associations with preterm birth and spontaneous abortion, but not with stillbirth.^11^ That review included studies with self-reported outcomes and the potential effects of confounding factors could not be examined because the estimates in the meta-analyses combined both unadjusted and adjusted estimates. Other outcomes, such as PROM, LBW and perinatal death, were not considered. As nucleic acid amplification tests (NAATs) for *M. genitalium* detection are increasingly used for widespread testing in populations including pregnant women, an updated review of the evidence about associations between *M. genitalium* and objectively documented adverse pregnancy and perinatal outcomes is warranted.

The primary objective of this study was to assess the association between *M. genitalium* infection in pregnancy and PTB. Secondary outcomes were spontaneous abortion, PROM, LBW and perinatal death.

## Methods

This systematic review and meta-analysis is registered in the PROSPERO database (CRD42016050962) and follows a published protocol, which also addresses *N. gonorrhoeae* and other genital mycoplasmas.^12^ We report our findings using the Preferred Reporting Items for Systematic reviews and Meta-Analyses 2020 (online supplemental table S1).^13^

10.1136/sextrans-2021-055352.supp1 Supplementary data

### Eligibility criteria

Studies reporting on *M. genitalium* during pregnancy, labour or the immediate postpartum period were eligible for inclusion if they reported on any of the following outcomes (in order of occurrence during pregnancy): spontaneous abortion, PROM (preterm and term), PTB, LBW, and perinatal or neonatal death. We included clinical trials, cohort, case–control and cross-sectional studies but excluded individual case reports, case series, opinion articles and studies without a comparison group.

### Information sources and search strategy

We searched Medline, Excerpta Medica database (EMBASE), the Cumulative Index to Nursing and Allied Health Literature (CINAHL) and the Cochrane library databases from 1948 to 11 August 2021. Search terms combined thesaurus and free-text terms for pregnancy and *M. genitalium* and the outcomes of interest. The search strategy is published^12^ and listed in online supplemental text S1. We examined reference lists of included studies for additional articles. The searches did not apply language restrictions, but we included only articles published in English or German (languages spoken fluently by review team members).

### Study selection and data extraction

One reviewer (LV) screened titles and abstracts (online supplemental text S2). Two reviewers (LV, DE-G) independently screened the full text of potentially relevant articles and extracted data independently into a standardised, piloted form in a Research Electronic Data Capture database (REDCap, Vanderbilt University, Tennessee, USA) recording study design, participant characteristics, presence or absence of *M. genitalium,* pregnancy, perinatal or neonatal outcomes, and other STI and genital infections. Standard definitions for outcomes were used,^12^ or if necessary, we used the definitions used by the authors. If results were described for more than one anatomical site, we used the following order of preference: vaginal or cervical swabs, urine, amniotic fluid, placenta because the original site of infection is the genital tract, with other sites reflecting increasingly distant sites of potential ascending infection. All diagnoses were made by NAATs. Discrepancies were resolved by discussion or by the decision of a third reviewer (NL, CF).

### Risk of bias in individual studies

Two reviewers assessed the risk of bias in each study independently (LV, DE-G or CF), using checklists published by the UK National Institute for Health and Care Excellence for case–control and cohort studies.^14^ A third reviewer resolved discrepancies (NL). Each study was assessed for internal and external validity overall as having all or most of checklist criteria fulfilled (++), some checklist criteria fulfilled (+) or few or no checklist criteria fulfilled (−), and the main sources of bias were recorded.

### Data synthesis and analysis

We used the ‘metan’ command in Stata (V.15.1; StataCorp, College Station, Texas, USA) for analyses. We used the OR as the measure of association for all study designs, on the assumption that the risk ratio and OR would be similar, as the outcomes of interest are usually rare events. We calculated the crude OR and its 95% CI based on raw data from the paper, or we extracted the published values if raw data were not available. If there were no events in one group, we applied a continuity correction, adding 0.5 to each cell. Where authors reported a multivariable analysis, we extracted the adjusted OR (aOR, with its 95% CI) and recorded the variables included in the model. We examined forest plots for each outcome, by study design, and used the I^2^ statistic to examine the level of variability in effect estimates due to heterogeneity between studies other than that due to chance.^15^

For outcomes reported by two or more studies, we used random-effects models for meta-analyses,^15^ based on an assessment of statistical and clinical heterogeneity. The random-effects model is appropriate for meta-analysis of observational studies because it assumes that there are differences between studies in the underlying effects because of heterogeneity in study populations and measurement of exposures and outcomes.^16^ We first examined estimates for cohort and case–control studies separately. Where appropriate, we estimated a summary OR (and 95% CI) and a prediction interval, which displays the expected range of effect estimates in future studies.^15^ For adjusted estimates, we used the same approach as for the unadjusted analyses. For outcomes for which there were at least two studies of the same design, we categorised study locations as high income and non-high income (combining low-income and middle-income countries), based on the 2019 World Bank list.^17^

### Risk of bias across studies and certainty of the body of evidence

We planned to examine publication bias by generating a funnel plot for outcomes reported by 10 or more studies. We did not conduct any subgroup analyses. We used the Grading of Recommendations Assessment, Development and Evaluation approach, adapted to assess the certainty of the evidence about the possible causal association^18^ between *M. genitalium* and each outcome.

## Results

The searches of electronic databases identified 116 records and we screened 104 records after exclusion of duplicates. Of 26 full-text articles assessed for eligibility (online supplemental figure S1), we included 10 studies, which reported on 18 outcomes (table 1, online supplemental table S2).^3 19–27^

**Table 1:** Summary of study characteristics of included studies

First author, publication year, reference number	Study design	Timing of specimen collection	Specimen type	Total enrolled, N	Sample size for outcome, events in women with *Mycoplasma genitalium/*total with the outcome, n/n (%)				
					PTB	PROM	LBW	SAB	PND
Agger, 2014^21^	Cohort	1st or 2nd trimester	Endocervical swab	783	676, 0/54 (0)	NR	NR	NR	NR
Averbach, 2013^19^	Cohort*	1st or 2nd trimester	Endocervical swab	100	66, 1/11 (9)	NR	81, 1/11 (9)	81, 1/9 (11)	NR
Choi, 2012^22^	Case–control	NR	Vaginal swab	217	217, 0/100 (0)	NR	NR	NR	NR
Edwards, 2006^23^	Cohort	NR	Not clear	137	134†	NR	NR	NR	NR
Hitti, 2010^3^	Case–control*	<48 hours post partum	Endocervical swab	1338	1328, 29/661 (4)	NR	NR	NR	NR
Kataoka, 2006^24^	Cohort	1st trimester	Vaginal swab	1040	871, 0/15 (0)	871, 0/7 (0)	NR	877, 0/5 (0)	872, 0/1 (0)
Labbe, 2002^20^	Case–control	<24 hours post partum	Endocervical swab	1014	799, 16/119 (13)	NR	NR	653, 2/53 (4)	725, 8/125 (6)
Oakeshott, 2004^25^	Cohort	1st trimester	Urine	1216	699, 0/39 (0)	NR	NR	894, 1/92 (1)	NR
Rahimkhani, 2018^27^	Cohort	1st or 2nd trimester	Urine	119	NR	NR	NR	119, 6/31 (19)	NR
Short, 2010^26^	Case–control*	NR	Urine	216	NR	NR	NR	213, 3/82 (4)	NR

*Authors reported both univariable and multivariable analyses.

†Numerator and denominator not reported in text. OR and 95% CI, as reported by authors, used in meta-analysis.

LBW, low birth weight; NR, not reported; PROM, premature rupture of membranes; PND, perinatal death; PTB, preterm birth; SAB, spontaneous abortion.

Study locations and sociodemographic information are reported in online supplemental table S3. Briefly, seven studies took place in high-income countries,^19 21–26^ and three in low/middle-income countries.^3 20 27^ Seven studies took place in urban locations.^3 19 22–26^ Age was reported in three studies,^19 25 27^ eight reported on ethnicity,^19–26^ four included smokers^3 19 23 26^ and two included women with multiple pregnancies.^3 21^ In three studies, authors reported adjusted ORs from multivariable analyses.^3 19 26^

The authors of seven studies reported timing of specimen collection: specimens were obtained during the first trimester in two studies,^24 25^ during the first or second trimester in three,^19 21 27^ and in the early postpartum period in two studies^3 20^ (table 1, online supplemental table S2). The sample types were endocervical swabs in four studies,^3 19–21^ urine in three^25–27^ and vaginal swabs in two studies.^22 24^ In one study, specimen type was unclear.^23^ In three studies, women who tested positive for an STI were given antibiotic treatment.^3 22 24^ In one study in Japan, the authors reported that they gave antibiotics if *C. trachomatis* and/or *N. gonorrhoeae* were detected but not for any *Mycoplasma* spp alone, that is, in the absence of *C. trachomatis* or *N. gonorrhoeae*.^24^ This was also the only study that reported the timing of antibiotic treatment (first or second trimester) (online supplemental table S4).

In all included studies, the authors tested for one or more other STI or genital infections (online supplemental tables S5–S8 report on coinfections with *M. genitalium* in included studies). *C. trachomatis* was tested for in all but one study^20^ and was detected in 2.2%–7.5% of women. *N. gonorrhoeae* was tested for in seven studies^3 19–24^ and detected in 0.0%–7.9% of women. In four studies, 0.0%–4.8% of women had positive serological tests for syphilis^19 20 22 23^ and in four studies, bacterial vaginosis was diagnosed in 0.8%–56.0% of women.^19 22 23 25^ In six studies, one or more of *M. hominis*, *Ureaplasma urealyticum*, *U. parvum*, *Trichomonas vaginalis*, herpes simplex virus type 2 or HIV were also reported^3 20–24^ (online supplemental table S9).

### Risk of bias

In case–control studies, the potential for selection bias could not be assessed because response rates for cases and controls were only reported in one study.^3^ In both case–control and cohort studies, the risk of confounding was high because potential confounding factors were often not reported and multivariable analyses were conducted in very few studies. Among the four case–control studies, all or most of checklist criteria (++) were completed for internal validity for two studies.^3 26^ For external validity, three of these studies had some checklist criteria (+) completed^3 20 22^ (online supplemental table S10). Five of the six cohort studies had some checklist criteria (+) completed for internal validity^19 21 23–25^ and two for external validity^21 23^ (online supplemental table S11). There were too few studies to assess publication bias using funnel plots for any outcome.

### Preterm birth

**Figure 1:**
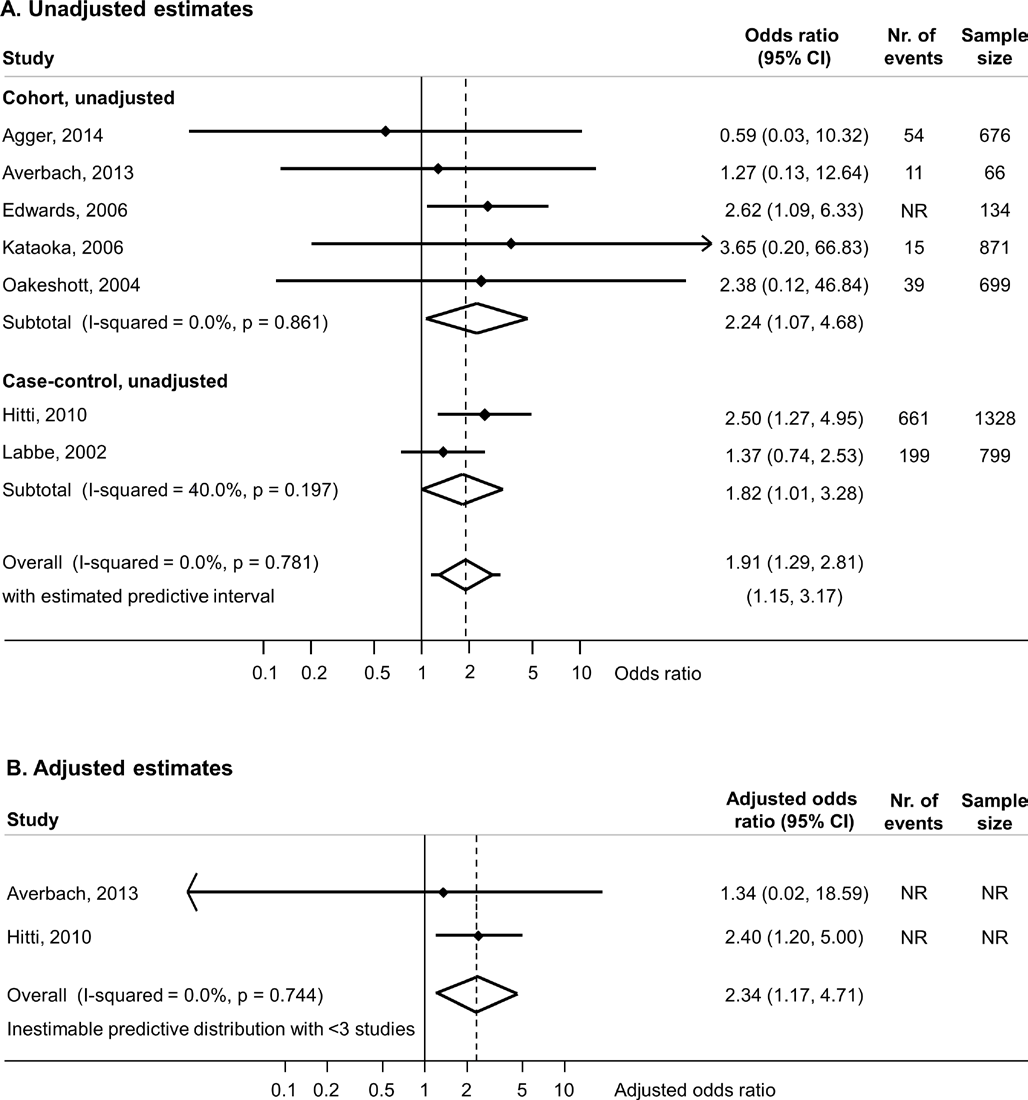
Random-effects meta-analysis of studies reporting on the association between *Mycoplasma genitalium* during pregnancy and preterm birth. Forest plots show effect estimates for each study for unadjusted estimates (A) and adjusted estimates (B). In studies reporting multivariable analyses, the numbers of events or total number of observations included were not reported (NR). For each study, the solid diamond is the point estimate, the lines either side are the 95% CIs. A line ending in an arrow means that the confidence limit lies beyond the values of the x-axis. The open diamond is the summary estimate. The lines either side of the open diamond show the prediction interval if there are three or more studies in the meta-analysis. The x-axis is on the log scale.

Eight of the ten included studies reported on *M. genitalium* and the primary outcome, PTB.^3 19–25^ One study was not included in meta-analysis because the authors reported that no woman tested positive for *M. genitalium* infection.^22^ Of the seven studies included in the meta-analysis of univariable results, five cohort studies reported on 2446 women,^19 21 23–25^ and two case–control studies reported on 2127 women.^3 20^ The meta-analysis of all seven studies found an OR 1.91 (95% CI 1.29 to 2.81, I^2^=0%) and increased odds of PTB in both cohort studies and case–control studies (figure 1A). Two studies reported the results of multivariable analyses. In the case–control study in Peru, age, cigarette smoking, second trimester bleeding, twin gestation and prior PTB were controlled for.^3^ In a cohort study in the USA, maternal age and history of preterm delivery were controlled for.^19^ The aOR in each study was similar to the unadjusted OR in the same study (figure 1A, B).^3 19^ The summary aOR was 2.34 (95% CI 1.17 to 4.71, I^2^=0%, 2 studies, table 2, figure 1B).

**Table 2:** Summary estimates for associations between *Mycoplasma genitalium* in pregnancy and adverse pregnancy and perinatal outcomes

Outcome, study design	Analysis	Number of studies, design	Sample size for outcome	I^2^ %	Summary OR (95% CI)
Preterm birth	Unadjusted, meta-analysis	7 5 cohort, 2 case–control	4573	0	1.91 (1.29 to 2.81)
	Adjusted, meta-analysis	2 1 cohort, 1 case–control	NR	0	2.34 (1.17 to 4.71)
Spontaneous abortion	Unadjusted, meta-analysis	6 4 cohort, 2 case–control	2837	0	1.00 (0.53 to 1.89)
	Adjusted	1 case–control	216	NA	0.9 (0.2 to 3.8)*
Premature rupture of membranes	Unadjusted	1 cohort	871	NA	7.62 (0.40 to 145.86)
Low birth weight	Unadjusted	1 cohort	81	NA	1.07 (0.02 to 10.39)
Perinatal death	Unadjusted	1 cohort	872	NA	38.42 (1.45 to 1021.43)
	Unadjusted	1 case–control	725	NA	1.07 (0.49 to 2.36)

*Adjusted OR reported to one decimal place, as in the publication, reference 25.

NA, not applicable; NR, not reported.

### Spontaneous abortion

**Figure 2:**
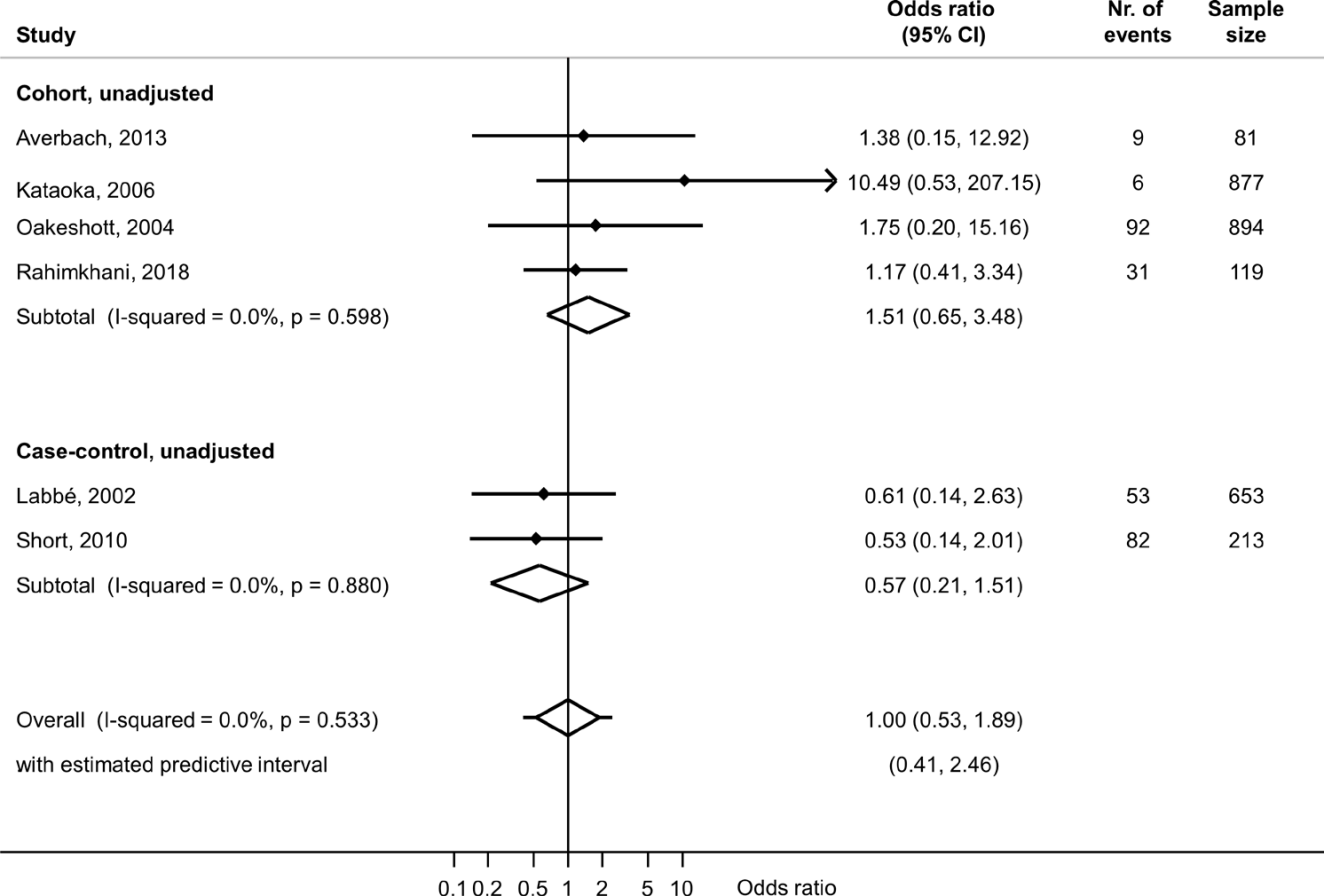
Random-effects meta-analysis of studies reporting an unadjusted association between *Mycoplasma genitalium* during pregnancy and spontaneous abortion. Forest plots show effect estimates for each study. For each study, the solid diamond is the point estimate, the lines either side are the 95% CIs. A line ending in an arrow means that the confidence limit lies beyond the values of the x-axis. The open diamond is the summary estimate. The lines either side of the open diamond show the prediction interval. The x-axis is on the log scale. Only one study reported a multivariable analysis (adjusted OR 0.90, 95% CI 0.2 to 3.8) (ref 26).

Six studies reported on associations with spontaneous abortion: four cohort studies including 1971 women^19 24 25 27^ and two case--control studies including 866 women.^20 26^ The summary unadjusted OR from all six studies was 1.00 (95% CI 0.53 to 1.89, I^2^=0%) (figure 2). Only one case–control study reported an aOR 0.9 (95% CI 0.2 to 3.8), adjusting for age, history of spontaneous abortion, smoking and gestational age^26^ (table 2).

### Premature rupture of membranes

One cohort study from Japan provided data about the univariable association between *M. genitalium* and PROM (OR 7.62, 95% CI 0.40 to 145.86, n=871) (table 2).^24^

### Low birth weight

One cohort study from the USA reported on the univariable association between *M. genitalium* and LBW (OR 1.07; 95% CI 0.02 to 10.39, n=81) (table 2).^19^

### Perinatal death

Two studies provided data about univariable associations between *M. genitalium* and perinatal death (online supplemental figure S2).^20 24^ In a case–control study in Guinea-Bissau, the OR was 1.07 (95% CI 0.49 to 2.36, n=725).^20^ In one cohort study in Japan, the OR was 38.42 (95% CI 1.45 to 1021.43, n=872).^26^ Owing to heterogeneity (I^2^=77%), we did not combine these estimates (table 2, online supplemental figure S2).

### Certainty of evidence

The certainty of evidence of causality (online supplemental table S12) was low for the outcomes preterm birth and spontaneous abortion, and very low for all other outcomes, based on assessment of study design and analysis.

## Discussion

This systematic review included 10 studies that reported on associations between *M. genitalium* and adverse pregnancy outcomes. For PTB, the summary unadjusted OR was 1.91 (95% CI 1.29 to 2.81, I^2^=0%, 7 studies) and summary aOR 2.34 (95% CI 1.17 to 4.71, I^2^=0%, 2 studies) with low between-study heterogeneity. For spontaneous abortion, the summary estimate of the unadjusted OR was 1.00 (95% CI 0.53 to 1.89, I^2^=0%, 6 studies). Only one study reported on the outcomes PROM, LBW and two reported on perinatal death.

### Strengths and weaknesses

Strengths of this systematic review are that we followed a protocol with a priori methods and we tried to reduce subjectivity by having two independent reviewers select studies for inclusion, extract data and assess the risk of bias. We examined adjusted effect estimates, where reported. It is important to report the confounder-adjusted estimate prominently, even when the data are sparse, because this is the most relevant measure for systematic reviews of observational studies that examine potential causal associations.^16^ We also examined findings from case–control and cohort studies separately, because the different study designs are subject to different biases.^28^ In this review, it made sense to combine the estimates for the outcomes PTB and spontaneous abortion because the strength of association in both was compatible. The main weakness of the review methods was that, despite a broad search strategy, we may have missed relevant studies in languages other than English or German. Given the small number of included studies, we could not assess the possibility of publication bias statistically.

### Comparison with other studies and interpretation

Our findings update and add to those of the systematic review by Lis *et al*.^11^ Despite a large increase in the availability of testing for *M. genitalium*, the number of published studies investigating associations with adverse pregnancy outcomes has not increased substantially since 2014, when the search of Lis *et al* ended. In contrast with Lis *et al*, we only included studies in which outcomes were directly observed, so there was no potential for recall bias in studies that rely on self-reported outcomes, and we examined the results of unadjusted and confounder-adjusted analyses separately. No new studies about the association between *M. genitalium* and PTB have been published since 2014. We found an association in meta-analysis of unadjusted estimates, and in the two studies with a multivariable analysis, the increased risk of preterm birth in women with *M. genitalium*, compared with those without, persisted.^3 19^ The potential for confounding cannot be assessed in detail; however, both studies adjusted for age, but not all potentially relevant confounders were considered. For spontaneous abortion, we included six studies and did not find evidence of an association with *M. genitalium* in univariable analyses, or in the only study reporting a multivariable analysis (aOR 0.9, 95% CI 0.2 to 3.8).^26^ In contrast, Lis *et al* reported an unadjusted summary OR of 1.82 (95% CI 1.10 to 3.03), but only one of the included studies did not use self-reported outcomes.^11^ The certainty of evidence for these outcomes is low because reliance on unadjusted findings means that the estimate from fully confounder-adjusted analyses might be substantially different. For all other outcomes, the evidence is very uncertain because of the paucity of studies.

The summary point estimates for the association between *M. genitalium* and PTB (case–control studies, OR 1.82; 95% CI 1. 01 to 3.28 and cohort studies, OR 2.24; 95% CI 1.07 to 4.68) appear higher than those obtained from systematic reviews of studies of associations with other bacterial STIs, although the overlap in CIs for all estimates means that the finding might be due to chance. For *C. trachomatis*, the reported summary unadjusted OR for preterm labour in case–control studies was 1.29 (95% CI 1.11 to 1.50, I^2^=82%, 10 studies) and for cohort studies 1.54 (95% CI 1.48 to 1.60, I^2^=98%, 13 studies).^5^ Tang *et al* 5 identified many more studies about *C. trachomatis* overall and found that summary confounder-adjusted ORs were lower than the unadjusted estimates. For *T. vaginalis*, the summary unadjusted risk ratio was 1.42 (95% CI 1.15 to 1.75, I^2^=63%, 9 studies)^29^ and for *N. gonorrhoeae,* the summary unadjusted OR was 1.55 (95% CI 1.21 to 1.99, 18 studies).^6^ Interpretation of the evidence overall is limited by poor reporting, partly because *M. genitalium* was not a primary study objective in many studies, with analyses done retrospectively using stored samples. Additionally, reporting of information about antibiotic treatment, the trimester in which treatment was given and coinfections was poor. Only 3 out of the 10 studies reported information on antibiotic treatment, and only 1 study specified the trimester in which treatment was given. We documented if studies tested for other STIs and if coinfections with STI were reported. Unfortunately, coinfections with STIs were often not reported, which made it difficult to assess if they were confounding variables.

### Implications for practice and research

This systematic review found some evidence that *M. genitalium* might increase the risk of PTB, but not spontaneous abortion. The limitations of the evidence available from published studies mean that there is a low level of certainty about these estimated effect sizes and there is insufficient evidence to determine whether *M. genitalium* is causally associated with PTB or other adverse pregnancy and perinatal outcomes. Future studies examining the association between *M. genitalium* infection and adverse pregnancy and birth outcomes are needed. These studies should be designed prospectively, with adequate statistical power to conduct multivariable analyses that control for potential confounding and should report on coinfections and provision and timing of antibiotic treatment during pregnancy. There are ongoing trials of the effectiveness of testing for STIs in pregnancy,^30 31^ in which the association between *M. genitalium* and the prespecified outcomes could be examined. Randomised controlled trials will be needed to determine whether an intervention to offer screening and treatment for *M. genitalium* in pregnancy reduces PTB or other adverse pregnancy outcomes. In view of the propensity for, and increasing levels of, antimicrobial resistance to azithromycin,^32^ testing and treatment for asymptomatic *M. genitalium* in pregnancy is not indicated at present.

**Key messages**

- Few studies have examined associations between *Mycoplasma genitalium* and adverse pregnancy and perinatal outcomes.
- There is some evidence of an association between *M. genitalium* in pregnancy and preterm birth, but no evidence of an association with spontaneous abortion.
- There is insufficient evidence to recommend screening and treatment for asymptomatic *M. genitalium* infection in pregnancy.

## Data Availability

All data are available in the manuscript or the online supplemental materials.
