# Supplementary material for: Adverse pregnancy and perinatal outcomes associated with Mycoplasma genitalium: systematic review and meta-analysis
Source: Sex Transm Infect. 2022 Mar 29;98(3):222–7. doi: 10.1136/sextrans-2021-055352 (PMC9016252; doi:10.1136/sextrans-2021-055352)
Supplement: Supplementary data [file sextrans-2021-055352supp001.pdf]

Online supplemental material

# Adverse pregnancy and perinatal outcomes associated with *Mycoplasma genitalium:* systematic review and meta-analysis

Carole Frenzer,[1] Dianne Egli-Gany,[1] Lisa M Vallely,[2,3] Andrew J Vallely,[2,3] Nicola Low [1]

1. Institute of Social and Preventive Medicine, University of Bern, Switzerland
2. Public Health Interventions Research Program, Kirby Institute, UNSW Sydney, Australia
3. Papua New Guinea Institute of Medical Research, Goroka, Papua New Guinea



Frenzer C, *et al. Sex Transm Infect* 2022; 98:222–227. doi: 10.1136/sextrans-2021-055352

Online supplemental material

**Contents**

Adverse pregnancy and perinatal outcomes associated with *Mycoplasma genitalium:* systematic review and meta-analysis ..... 1

Text S1: Search strategy ..... 1

Text S2: Exclusion criteria at first stage ..... 2

Table S1: Preferred reporting items for systematic reviews and meta-analysis (PRISMA 2020 item checklist) ..... 3

Table S2: Descriptive characteristics of included studies ..... 6

Table S3: Income group and socio-demographic characteristics in included studies ..... 7

Table S4: Reported antibiotic treatment in included studies ..... 8

Table S5: Overview of *C. trachomatis* infections and co-infections in study populations in included studies ..... 9

Table S6: Overview of *N. gonorrhoeae* infections and co-infections in study populations in included studies ..... 10

Table S7: Overview of *T. pallidum* infections and co-infections in study populations in included studies ..... 11

Table S8: Overview of bacterial vaginosis and co-infections in study populations in included studies ..... 12

Table S9: Overview of overall positivity of genital infections in study populations in included studies[1] ..... 13

Table S10: Risk of bias assessment, case control studies ..... 14

Table S11: Risk of bias assessment, cohort studies ..... 15

Table S12: Summary of findings table for studies examining the association between *Mycoplasma genitalium* and adverse pregnancy outcomes ..... 17

Figure S1: Flow chart of identified and selected studies for inclusion ..... 18

Figure S2: Forest plot of unadjusted effect sizes for association between *M. genitalium* during pregnancy and perinatal death ..... 19

Codebook S1, study and population characteristics, appended ..... 20

Codebook S2, Mycoplasma genitalium variables, appended ..... 20

Online supplemental material

**Text S1: Search strategy**

| | |
|---|---|
| 1. Terms for population | "pregnancy" or "prenatal" or "antenatal" |
| 2. Terms for exposure | "*Mycoplasma genitalium*" |
| 3. Terms for outcomes | "birth outcome" or "adverse birth outcome" or "adverse pregnancy outcome" or "perinatal morbidity" or "perinatal mortality" or "perinatal outcome" or "premature birth" or "premature delivery" or "very preterm birth" or "preterm birth" or "preterm delivery" or "premature labour" or "preterm labour" or "premature labor" or "preterm labor" or "premature rupture of membranes" or "preterm rupture of membranes" or "preterm premature rupture of membranes" or "low birth weight" or "intrauterine growth retardation" or "intrauterine growth restriction" or "small for gestational age" or "gestational age" or "stillbirth" or "perinatal mortality" or "perinatal morbidity" or "perinatal death" or "neonatal mortality" or "neonatal morbidity" or "neonatal death" or "fetal death" or "miscarriage" or "spontaneous abortion" or "chorioamnionitis" |
| 4. Search = #1 + # 2 + # 3 | |



Frenzer C, *et al. Sex Transm Infect* 2022; 98:222–227. doi: 10.1136/sextrans-2021-055352

Online supplemental material

**Text S2: Exclusion criteria at first stage**

If the title mentions one of the following without reference to pregnancy, sexually transmitted infections or *M. genitalium* the article was excluded in the first stage of the screening process:

- Sexual assault
- Algorithm
- Infertility
- Contraception/ Family planning
- Ectopic/tubal pregnancy
- UTI in women
- Gonococcal arthritis
- Gynecology/gynaecology
- Induced abortion
- Syphilis (only)
- Trachomatis (only)
- Chlamydia (only)
- Treatment guidelines/ treatment schedules
- Anti-retroviral therapy
- Tetanus
- Sexual health
- Child sex abuse
- Polio

If the article was found to be a case report, review article or letter, the article was excluded at any stage of the review process.



Frenzer C, *et al. Sex Transm Infect* 2022; 98:222–227. doi: 10.1136/sextrans-2021-055352

Online supplemental material

**Table S1: Preferred reporting items for systematic reviews and meta-analysis (PRISMA 2020 item checklist)**

| Section/topic | # | Checklist item | Section and paragraph |
|---|---|---|---|
| Title | 1 | Identify the report as a systematic review. | Title |
| Abstract | 2 | See the PRISMA 2020 for Abstracts checklist. | Abstract includes as many items as allowed in word count |
| Rationale | 3 | Describe the rationale for the review in the context of existing knowledge. | Introduction |
| Objectives | 4 | Provide an explicit statement of the objective(s) or question(s) the review addresses. | Introduction, para 3 |
| Eligibility criteria | 5 | Specify the inclusion and exclusion criteria for the review and how studies were grouped for the syntheses. | Methods, Eligibility criteria; Text S2 |
| Information sources | 6 | Specify all databases, registers, websites, organisations, reference lists and other sources searched or consulted to identify studies. Specify the date when each source was last searched or consulted. | Methods, Information sources |
| Search strategy | 7 | Present the full search strategies for all databases, registers and websites, including any filters and limits used. | Text S1 |
| Selection process | 8 | Specify the methods used to decide whether a study met the inclusion criteria of the review, including how many reviewers screened each record and each report retrieved, whether they worked independently, and if applicable, details of automation tools used in the process. | Methods, Study selection; Text S2 |
| Data collection process | 9 | Specify the methods used to collect data from reports, including how many reviewers collected data from each report, whether they worked independently, any processes for obtaining or confirming data from study investigators, and if applicable, details of automation tools used in the process. | Methods, Study selection and data extraction |
| Data items | 10a | List and define all outcomes for which data were sought. Specify whether all results that were compatible with each outcome domain in each study were sought (e.g. for all measures, time points, analyses), and if not, the methods used to decide which results to collect. | Methods, Study selection and data extraction; Protocol, Codebook S2 |
| | 10b | List and define all other variables for which data were sought (e.g. participant and intervention characteristics, funding sources). Describe any assumptions made about any missing or unclear information. | Protocol, Codebook S1 |
| Study risk of bias assessment | 11 | Specify the methods used to assess risk of bias in the included studies, including details of the tool(s) used, how many reviewers assessed each study and whether they worked independently, and if applicable, details of automation tools used in the process. | Methods, Risk of bias in individual studies |
| Effect measures | 12 | Specify for each outcome the effect measure(s) (e.g. risk ratio, mean difference) used in the synthesis or presentation of results. | Methods, Data synthesis and analysis |

Online supplemental material

| | | | |
|---|---|---|---|
| Synthesis methods | 13a | Describe the processes used to decide which studies were eligible for each synthesis (e.g. tabulating the study intervention characteristics and comparing against the planned groups for each synthesis (item #5)). | Methods, Data synthesis and analysis |
| | 13b | Describe any methods required to prepare the data for presentation or synthesis, such as handling of missing summary statistics, or data conversions. | Methods, Data synthesis and analysis |
| | 13c | Describe any methods used to tabulate or visually display results of individual studies and syntheses. | Methods, Data synthesis and analysis |
| | 13d | Describe any methods used to synthesize results and provide a rationale for the choice(s). If meta-analysis was performed, describe the model(s), method(s) to identify the presence and extent of statistical heterogeneity, and software package(s) used. | Methods, Data synthesis and analysis |
| | 13e | Describe any methods used to explore possible causes of heterogeneity among study results (e.g. subgroup analysis, meta-regression). | Risk of bias across studies and certainty of the body of evidence |
| | 13f | Describe any sensitivity analyses conducted to assess robustness of the synthesized results. | Risk of bias across studies and certainty of the body of evidence |
| Reporting bias assessment | 14 | Describe any methods used to assess risk of bias due to missing results in a synthesis (arising from reporting biases). | Risk of bias across studies and certainty of the body of evidence |
| Certainty assessment | 15 | Describe any methods used to assess certainty (or confidence) in the body of evidence for an outcome. | Risk of bias across studies and certainty of the body of evidence |
| Study selection | 16a | Describe the results of the search and selection process, from the number of records identified in the search to the number of studies included in the review, ideally using a flow diagram. | Results ,para 1-2, p. 6, Figure S1 |
| | 16b | Cite studies that might appear to meet the inclusion criteria, but which were excluded, and explain why they were excluded. | Not done |
| Study characteristics | 17 | Cite each included study and present its characteristics. | Results, Table 1; Table S2 |
| Risk of bias in studies | 18 | Present assessments of risk of bias for each included study. | Tables S10 and S11 |
| Results of individual studies | 19 | For all outcomes, present, for each study: (a) summary statistics for each group (where appropriate) and (b) an effect estimate and its precision (e.g. confidence/credible interval), ideally using structured tables or plots. | Results, Table 2; Figure 1; Figure 2; Figure S2 |
| Results of syntheses | 20a | For each synthesis, briefly summarise the characteristics and risk of bias among contributing studies. | Results, named paragraph for each outcome |

Online supplemental material

| | | | |
|---|---|---|---|
| | 20b | Present results of all statistical syntheses conducted. If meta-analysis was done, present for each the summary estimate and its precision (e.g. confidence/credible interval) and measures of statistical heterogeneity. If comparing groups, describe the direction of the effect. | Results, Table 2; Figure 1; Figure 2; Figure S2 |
| | 20c | Present results of all investigations of possible causes of heterogeneity among study results. | Results, Risk of bias |
| | 20d | Present results of all sensitivity analyses conducted to assess the robustness of the synthesized results. | Not applicable |
| Reporting biases | 21 | Present assessments of risk of bias due to missing results (arising from reporting biases) for each synthesis assessed. | Not done |
| Certainty of evidence | 22 | Present assessments of certainty (or confidence) in the body of evidence for each outcome assessed. | Results, Certainty of evidence; Table S12 |
| Discussion | 23a | Provide a general interpretation of the results in the context of other evidence. | Discussion, Comparison with other studies and interpretation |
| | 23b | Discuss any limitations of the evidence included in the review. | Discussion, Comparison with other studies and interpretation |
| | 23c | Discuss any limitations of the review processes used. | Discussion, Strengths and weaknesses |
| | 23d | Discuss implications of the results for practice, policy, and future research. | Discussion, Implications for practice and research |
| Registration and protocol | 24a | Provide registration information for the review, including register name and registration number, or state that the review was not registered. | After Abstract |
| | 24b | Indicate where the review protocol can be accessed, or state that a protocol was not prepared. | Methods, para 1 |
| | 24c | Describe and explain any amendments to information provided at registration or in the protocol. | Not applicable |
| Support | 25 | Describe sources of financial or non-financial support for the review, and the role of the funders or sponsors in the review. | After main text |
| Competing interests | 26 | Declare any competing interests of review authors. | After main text |
| Availability of data, code and other materials | 27 | Report which of the following are publicly available and where they can be found: template data collection forms; data extracted from included studies; data used for all analyses; analytic code; any other materials used in the review. | Online supplemental material |

Online supplemental material

**Table S2: Descriptive characteristics of included studies**

| First author, publication year | Assessment of gestational age | Timing of specimen collection | Specimen type | Total number enrolled | Sample size for outcome | Outcome+ MG+ | Outcome+ MG- | Outcome- MG+ | Outcome- MG- | Outcome | Outcome definition |
|---|---|---|---|---|---|---|---|---|---|---|---|
| **Agger, 2014** | NR | 1st or 2nd trimester | Endocervical swab | 783 | 676 | 0 | 54 | 9 | 613 | PTB | < 37 weeks |
| **Averbach, 2013** | USS, LMP | 1st or 2nd trimester | Endocervical swab | 100 | 66 | 1 | 10 | 4 | 51 | PTB | 24-36 weeks |
| | | | | 100 | 81 | 1 | 10 | 6 | 64 | LBW | < 2500g |
| | | | | 100 | 81 | 1 | 8 | 6 | 66 | SAB | NR |
| **Choi, 2012** | NR | NR | Vaginal swab | 217 | 191 | 0 | 100 | 0 | 91 | PTB | NR |
| **Edwards, 2006** | NR | NR | Not clear | 137 | 134 | NR | NR | NR | NR | PTB | < 37 weeks |
| **Hitti, 2010** | USS, LMP, NN | <48 hours post-partum | Endocervical swab | 1338 | 1328 | 29 | 632 | 12 | 655 | PTB | 20-36 weeks |
| **Kataoka, 2006** | USS, LMP | 1st trimester | Vaginal swab | 1040 | 871 | 0 | 15 | 7 | 849 | PTB | < 34 weeks[1] |
| | | | | 1040 | 871 | 0 | 7 | 7 | 857 | PROM | NR |
| | | | | 1040 | 877 | 0 | 5 | 7 | 865 | SAB | NR |
| | | | | 1040 | 872 | 0 | 1 | 7 | 864 | PND | NR |
| **Labbé, 2002** | NR | <24 hours post-partum | Endocervical swab | 1014 | 799 | 16 | 183 | 36 | 564 | PTB | < 37 weeks |
| | | | | 1014 | 653 | 2 | 51 | 36 | 564 | SAB | < 20 weeks |
| | | | | 1014 | 725 | 8 | 117 | 36 | 564 | PND | > 20 weeks |
| **Oakeshott, 2004** | LMP | 1st trimester | Urine | 1216 | 699 | 0 | 39 | 3 | 657 | PTB | < 37 weeks |
| | | | | 1216 | 894 | 1 | 91 | 5 | 797 | SAB | <16 weeks |
| **Rahimkhani, 2018** | NR | 1st or 2nd trimester | Urine | 119 | 119 | 6 | 25 | 15 | 73 | SAB | NR |
| **Short, 2010** | NR | NR | Urine | 216 | 213 | 3 | 79 | 9 | 125 | SAB | < 22 weeks |

**Abbreviations:** LBW, low birth weight; LMP, last menstrual period; NN, neonatal; NR, not reported; PROM, premature rupture of membranes; PND perinatal death; PTB, preterm birth; SAB, spontaneous abortion; USS, ultrasound scan.

[1] Included spontaneous abortion (n=5 at 11-15 weeks) and intrauterine death (n=1 at 24 weeks). These outcomes were extracted separately

Online supplemental material

**Table S3: Income group and socio-demographic characteristics in included studies**

| First author, year | Country of study | World bank classification | Urban or rural location | Age in years (mean(SD)/median (IQR)/min-max) | Ethnic group/racial categories/nationality | Smokers included (%) | Multiple pregnancies |
|---|---|---|---|---|---|---|---|
| **Agger, 2014** | USA | High income | Mixed | NR[1] | Mixed | NR | Yes[2] (5/783) |
| **Averbach, 2013** | USA | High income | Urban | NR/25.0 (22.0-30.0)/NR | Mixed | Yes (11.6%) | No |
| **Choi, 2012** | South Korea | High income | Urban | NR | Asian | NR | NR |
| **Edwards, 2006** | USA | High income | Urban | NR | Mixed | Yes (15.67%) | No |
| **Hitti, 2010** | Peru | Non-high income | Urban | NR[3] | NR | Yes (6.48%) | Yes (73/1328) |
| **Kataoka, 2006** | Japan | High income | Urban | NR | Asian | NR | No |
| **Labbé, 2002** | Guinea-Bissau | Non-high income | NR | NR | Black | NR | NR |
| **Oakeshott, 2004** | United Kingdom | High income | Urban | 31 (NR)/NR/16-48 | Mixed | NR | NR |
| **Rahimkhani, 2018** | Iran | Non-high income | NR | 29 (NR)/NR/NR | NR | NR | NR |
| **Short, 2010** | USA | High income | Urban | NR[4] | Mixed | Yes (34.72%) | NR |

**Abbreviations:** IQR, interquartile range; NR, not reported; SD, standard deviation

[1] Ages only available for subgroups
[2] Multiple Pregnancies were excluded from the analysis
[3] Ages only available for subgroups
[4] Ages only available for subgroups



Frenzer C, *et al. Sex Transm Infect* 2022; 98:222–227. doi: 10.1136/sextrans-2021-055352

Online supplemental material

**Table S4: Reported antibiotic treatment in included studies**

| First author, year | Antibiotic treatment provided | Timing of antibiotic treatment |
|---|---|---|
| Agger, 2014 | Yes, some positive women | NR |
| Averbach, 2013 | NR | NR |
| Choi, 2012 | NR | NR |
| Edwards, 2006 | NR | NR |
| Hitti, 2010 | Yes, some positive women | NR |
| Kataoka, 2006 | Yes, some positive women[1] | 1st or 2nd trimester |
| Labbé, 2002 | NR | NR |
| Oakeshott, 2004 | NR | NR |
| Rahimkhani, 2018 | NR | NR |
| Short, 2010 | NR | NR |

**Abbreviations:** NR, not reported

[1] Antibiotics were administered to women in whom *C. trachomatis* and/or *N. gonorrhoeae* was detected but not to those in whom any mycoplasma was detected in the absence of *C. trachomatis* or *N. gonorrhoeae*



Frenzer C, *et al. Sex Transm Infect* 2022; 98:222–227. doi: 10.1136/sextrans-2021-055352

Online supplemental material

**Table S5: Overview of *C. trachomatis* infections and co-infections in study populations in included studies**

| First author, year | All CT+[1] | CT+ in MG+[2] | CT+ in MG-[3] |
|---|---|---|---|
| Agger, 2014 | 33/676 (4.9%) | NR | NR |
| Averbach, 2013 | 6/94 (6.4%) | 1/8 (12.5%) | 5/86 (5.8%) |
| Choi, 2012 | 3/126 (2.4%) | NR | NR |
| Edwards, 2006 | 10/134 (7.5%) | NR | NR |
| Hitti, 2010 | 98/1328 (7.4%) | 9/41 (22.0%) | 89/1287 (6.9%) |
| Kataoka, 2006 | 28/877 (3.2%) | NR | NR |
| Labbé, 2002 | NR | NR | NR |
| Oakeshott, 2004 | 20/914 (2.2%) | 0/20 (0.0%) | 20/894 (2.2%) |
| Rahimkhani, 2018 | 8/119 (6.7%) | NR | NR |
| Short, 2010 | 15/216 (6.9%) | NR | NR |

**Abbreviations:** CT, *C. trachomatis*; MG, *M. genitalium*; NR, not reported

[1] Total number of participants tested positive for CT/total number of participants tested for this infection
[2] Total number of participants tested positive for CT and MG/total number of participants tested positive for MG
[3] Total number of participants tested positive for CT and negative for MG/total number of participants tested negative for MG



Frenzer C, *et al. Sex Transm Infect* 2022; 98:222–227. doi: 10.1136/sextrans-2021-055352

Online supplemental material

**Table S6: Overview of *N. gonorrhoeae* infections and co-infections in study populations in included studies**

| First author, year | All NG+[1] | NG+ in MG+[2] | NG+ in MG-[3] |
|---|---|---|---|
| Agger, 2014 | 7/676 (1.0%) | NR | NR |
| Averbach, 2013 | 1/94 (1.1%) | 0/8 (0.0%) | 1/86 (1.2%) |
| Choi, 2012 | 0/126 (0.0%) | NR | NR |
| Edwards, 2006 | 1/134 (0.7%) | NR | NR |
| Hitti, 2010 | 1/1328 (0.1%) | 0/41 (0.0%) | 1/1287 (0.1%) |
| Kataoka, 2006 | 1/877 (0.1) | NR | NR |
| Labbé, 2002 | 78/986 (7.9%) | 6/63 (9.5%) | 72/923 (7.8%) |
| Oakeshott, 2004 | NR | NR | NR |
| Rahimkhani, 2018 | NR | NR | NR |
| Short, 2010 | NR | NR | NR |

**Abbreviations:** NG, *N. gonorrhoeae*; MG, *M. genitalium*; NR, not reported

[1] Total number of participants tested positive for NG/total number of participants tested for this infection
[2] Total number of participants tested positive for NG and MG/total number of participants tested positive for MG
[3] Total number of participants tested positive for NG and negative for MG/total number of participants tested negative for MG



Frenzer C, *et al. Sex Transm Infect* 2022; 98:222–227. doi: 10.1136/sextrans-2021-055352

Online supplemental material

**Table S7: Overview of *T. pallidum* infections and co-infections in study populations in included studies**

| First author, year | All TP+[1] | TP+ in MG+[2] | TP+ in MG-[3] |
|---|---|---|---|
| Agger, 2014 | NR | NR | NR |
| Averbach, 2013 | 1/95 (1.1%) | 0/8 (0.0%) | 1/86 (1.2%) |
| Choi, 2012 | 0/126 (0.0%) | NR | NR |
| Edwards, 2006 | 0/134 (0.0%) | NR | NR |
| Hitti, 2010 | NR | NR | NR |
| Kataoka, 2006 | NR | NR | NR |
| Labbé, 2002 | 49/1014 (4.8%) | 4/63 (6.3%) | 45/951 (4.7%) |
| Oakeshott, 2004 | NR | NR | NR |
| Rahimkhani, 2018 | NR | NR | NR |
| Short, 2010 | NR | NR | NR |

**Abbreviations:** MG, *M. genitalium*; NR, not reported; TP, *T. pallidum*

Online supplemental material

**Table S8: Overview of bacterial vaginosis and co-infections in study populations in included studies**

| First author, year | All BV[1] | BV+ in MG+[2] | BV+ in MG-[3] |
|---|---|---|---|
| Agger, 2014 | NR | NR | NR |
| Averbach, 2013 | 42/75 (56.0%) | 6/8 (75%) | 36/67 (53.7%) |
| Choi, 2012 | 1/126 (0.8%) | NR | NR |
| Edwards, 2006 | 18/134 (13.4%) | NR | NR |
| Hitti, 2010 | NR | NR | NR |
| Kataoka, 2006 | NR | NR | NR |
| Labbé, 2002 | NR | NR | NR |
| Oakeshott, 2004 | 128/859 (14.9%) | 3/128 (2.3%) | 125/731 (17.1%) |
| Rahimkhani, 2018 | NR | NR | NR |
| Short, 2010 | NR | NR | NR |

**Abbreviations:** BV, bacterial vaginosis; MG, *M. genitalium*; NR, not reported

[1] Total number of participants tested positive for BV/total number of participants tested for this infection
[2] Total number of participants tested positive for BV and MG/total number of participants tested positive for MG
[3] Total number of participants tested positive for BV and negative for MG/total number of participants tested negative for MG

Online supplemental material

**Table S9: Overview of overall positivity of genital infections in study populations in included studies[1]**

| First author, year | All *M. hominis*[2] | All *U. urealyticum*[3] | All *U. parvum*[4] | All *T. vaginalis*[5] | All herpes[6] | All HIV[7] |
|---|---|---|---|---|---|---|
| Agger, 2014 | 119/676 (17.6%) | 50/676 (7.4%) | 331/676 (49.0%) | NR | 34/676 (5.0%) | NR |
| Averbach, 2013 | NR | NR | NR | NR | NR | NR |
| Choi, 2012 | 16/126 (12.7%) | 79/126 (62.7%) | NR | NR | NR | NR |
| Edwards, 2006 | NR | NR | NR | 10/134 (7.5%) | NR | NR |
| Hitti, 2010 | NR | NR | NR | 33/1328 (2.5%) | NR | NR |
| Kataoka, 2006 | 98/877 (11.2%) | 76/877 (8.7%) | 456/877 (52.0%) | NR | NR | NR |
| Labbé, 2002 | NR | NR | NR | 194/884 (22.0%) | NR | 95/1011 (9.4%) |
| Oakeshott, 2004 | NR | NR | NR | NR | NR | NR |
| Rahimkhani, 2018 | NR | NR | NR | NR | NR | NR |
| Short, 2010 | NR | NR | NR | NR | NR | NR |

**Abbreviations:** HIV, human immunodeficiency virus; *M. hominis*, *Mycoplasma hominis*; NR, not reported; *T. vaginalis*, *Trichomonas vaginalis*; *U. parvum*, *Ureaplasma parvum; U. urealyticum, Ureaplasma urealyticum*

[1] Prevalence of *C. trachomatis*, *N. gonorrhoeae*, *T. pallidum* and bacterial vaginosis is listed in table 2 to 5 in the thesis under all CT+, all NG+, all TP+ and all BV+
[2] Total number of participants tested positive for *M. hominis*/total number of participants tested for this infection
[3] Total number of participants tested positive for *U. urealyticum*/total number of participants tested for this infection
[4] Total number of participants tested positive for *U. parvum*/total number of participants tested for this infection
[5] Total number of participants tested positive for *T. vaginalis*/total number of participants tested for this infection
[6] Total number of participants tested positive for herpes/total number of participants tested for this infection
[7] Total number of participants tested positive for HIV/total number of participants tested for this infection



Frenzer C, *et al. Sex Transm Infect* 2022; 98:222–227. doi: 10.1136/sextrans-2021-055352

Online supplemental material

**Table S10: Risk of bias assessment, case control studies**

| Assessment criteria | Choi 2012 | Hitti 2010 | Labbé 2002 | Short 2010 |
|---|---|---|---|---|
| Appropriate and clearly focused question. | WC | WC | AA | WC |
| The cases and controls are taken from comparable populations. | NR | AA | AA | AA |
| The same exclusion criteria are used for both cases and controls. | NAD | AA | NAD | AA |
| What was the participation rate for each group (cases)? | NA | 98.7%[1] | NA | NA |
| What was the participation rate for each group (controls)? | NA | 99.9%[2] | NA | NA |
| Both groups are compared to establish their similarities or differences. | PA | WC | NAD | WC |
| Cases are clearly defined and differentiated from controls. | WC | WC | WC | WC |
| It is clearly established that controls are not cases. | WC | WC | WC | WC |
| Measures taken to prevent knowledge of primary exposure from influencing case ascertainment. | NA | NA | NA | NA |
| Exposure status is measured in a standard, valid and reliable way. | WC | WC | WC | WC |
| Main potential confounders are accounted for in design/analysis. | NAD | AA | NAD | WC |
| Confidence intervals provided? | No | Yes | Yes | Yes |
| Study results internally valid? | + | ++ | + | ++ |
| Study results externally valid? | + | + | + | - |

**Abbreviations:** AA, adequately addressed; NA, not applicable; NAD, not addressed; NR, not reported; PA, poorly addressed; WC, well covered; ++, all or most of checklist criteria fulfilled; + some of checklist criteria fulfilled; - few or no checklist criteria fulfilled

[1] Data were omitted for 5 cases with gestational age < 20 weeks or no documentation of gestational age assessment, 2 cases with higher-order multiple gestations and 2 additional subject who did not have cervical samples collected for *M. genitalium*.
[2] Data were omitted for 1 case who did not have cervical samples collected for *M. genitalium*.



Frenzer C, *et al. Sex Transm Infect* 2022; 98:222–227. doi: 10.1136/sextrans-2021-055352

Online supplemental material

**Table S11: Risk of bias assessment, cohort studies**

| Assessment criteria | Agger 2014 | Averbach 2013 | Edwards 2006 | Kataoka 2006 | Oakeshott 2004 | Rahimkhani 2018 |
|---|---|---|---|---|---|---|
| The method of allocation to exposure groups was unrelated to potential confounding factors. | NA | NA | NA | NA | NA | NA |
| Attempts made within design or analysis to balance both groups for potential confounders. | Yes | Yes | Yes | Yes | No | No |
| The groups were comparable at baseline, including all major confounding factors. | No | No | No | No | Unclear | Unclear |
| Based on above answers, was selection bias present? | High | High | High | High | Unclear | Unclear |
| If so, what is the likely direction of its effect? | Unclear | Unclear | Unclear | Unclear | Unclear | Unclear |
| The comparison groups received the same care and support apart from the exposure(s) studied. | Unclear | Yes | Unclear | Unclear | Unclear | Unclear |
| Participants receiving care and support were kept “blind” to intervention allocation. | NA | NA | NA | NA | NA | NA |
| Individuals administering care and support were kept “blind” to intervention allocation. | NA | NA | NA | NA | NA | NA |
| Based on above answers, was performance bias present? | Unclear | Low | Unclear | Unclear | Unclear | Unclear |
| If so, what is the likely direction of its effect? | Unclear | NA | Unclear | Unclear | Unclear | Unclear |
| All groups followed up for an equal length of time? | Yes | Yes | Yes | Yes | Yes | Yes |
| Number of participants who did not complete the intervention in each group? | NA | NA | NA | NA | NA | NA |
| The groups were comparable for intervention completion. | NA | NA | NA | NA | NA | NA |

Online supplemental material

| **Assessment criteria** | **Agger 2014** | **Averbach 2013** | **Edwards 2006** | **Kataoka 2006** | **Oakeshott 2004** | **Rahimkhani 2018** |
|---|---|---|---|---|---|---|
| For how many participants were no outcome data available? | 107/783 (13.7%) | 14/95 (14.7%) | 3/137 (2.2%) | 148/1040 (14.2%) | 301/1216 (24.8%) | 0/119 (0.0%) |
| Were groups comparable for outcome data? (there were no important or systematic differences between groups in terms of those who did not complete the intervention) | Unclear | Unclear | Unclear | Unclear | Unclear | Unclear |
| Based on above answers, was attrition bias present? | Unclear | Unclear | Low | Unclear | Unclear | Unclear |
| If so, what is the likely direction of its effect? | Unclear | Unclear | NA | Unclear | Unclear | Unclear |
| The study had an appropriate length of follow-up. | Yes | Yes | Yes | Yes | Yes | Yes |
| The study used a precise definition of outcome. | Yes | Yes | Yes | No | Yes | No |
| A valid, reliable method used to determine the outcome? | Unclear | Yes | Unclear | Yes | No | Unclear |
| Investigators were kept "blind" to participants' exposure to the intervention. | NA | NA | NA | NA | NA | NA |
| Investigators were kept "blind" to other important confounding factors. | NA | NA | NA | NA | NA | NA |
| Based on above answers, was detection bias present? | Unclear | Low | Unclear | Low | High | Unclear |
| If so, what is the likely direction of its effect? | Unclear | NA | Unclear | NA | Unclear | Unclear |
| Overall assessment of internal validity | + | + | + | + | + | - |
| Overall assessment of external validity | + | - | + | - | - | - |

**Abbreviations:** AO, adverse outcomes; High, high risk of bias; Low, low risk of bias; NA, not applicable; NK, not known; STI, sexually transmitted infections; Unclear, unclear of risk of bias; ++, all or most of checklist criteria fulfilled; + some of checklist criteria fulfilled; - few or no checklist criteria fulfilled .

Online supplemental material

**Table S12: Summary of findings table for studies examining the association between *Mycoplasma genitalium* and adverse pregnancy outcomes**

| Outcomes | Relative effect (95% CI) | Number of participants (studies) | Certainty of evidence (GRADE)[a] |
|---|---|---|---|
| Preterm birth (PTB) | OR 1.91 (1.29 to 2.81) | 4573 (5 cohort, 2 case-control studies) | ⊕⊕◯◯ Low[b] |
| Spontaneous abortion (SAB) | OR 1.00 (0.53 to 1.89) | 2837 (4 cohort, 2 case-control studies) | ⊕⊕◯◯[c] Low |
| Premature rupture of membranes (PROM) | OR 7.62 (0.40 to 145.86) | 871 (1 cohort study) | ⊕◯◯◯ Very low[d] |
| Low birth weight (LBW) | OR 1.07 (0.02 to 10.39) | 81 (1 cohort study) | ⊕◯◯◯ Very low[d] |
| Perinatal death (PND) | Not estimated[e] | 1597 (1 cohort, 1 case-control study) | ⊕◯◯◯ Very low[d] |

CI, confidence interval; OR, odds ratio.

[a] Grading of Recommendations, Assessment, Development and Evaluation (GRADE) Working Group grades of evidence
High certainty: we are very confident that the true effect lies close to that of the estimate of the effect.
Moderate certainty: we are moderately confident in the effect estimate: the true effect is likely to be close to the estimate of the effect, but there is a possibility that it is substantially different.
Low certainty: our confidence in the effect estimate is limited: the true effect may be substantially different from the estimate of the effect.
Very low certainty: we have very little confidence in the effect estimate: the true effect is likely to be substantially different from the estimate of effect.

[b] Only two studies control for confounding, high risk of selection bias;
[c] Only one study controlled for confounding; high risk of selection bias;
[d] No study controlled for confounding, imprecise estimates owing to small number of studies;
[e] Effect estimates not combined, owing to heterogeneity.



Frenzer C, *et al. Sex Transm Infect* 2022; 98:222–227. doi: 10.1136/sextrans-2021-055352

Online supplemental material

**Figure S1: Flow chart of identified and selected studies for inclusion**

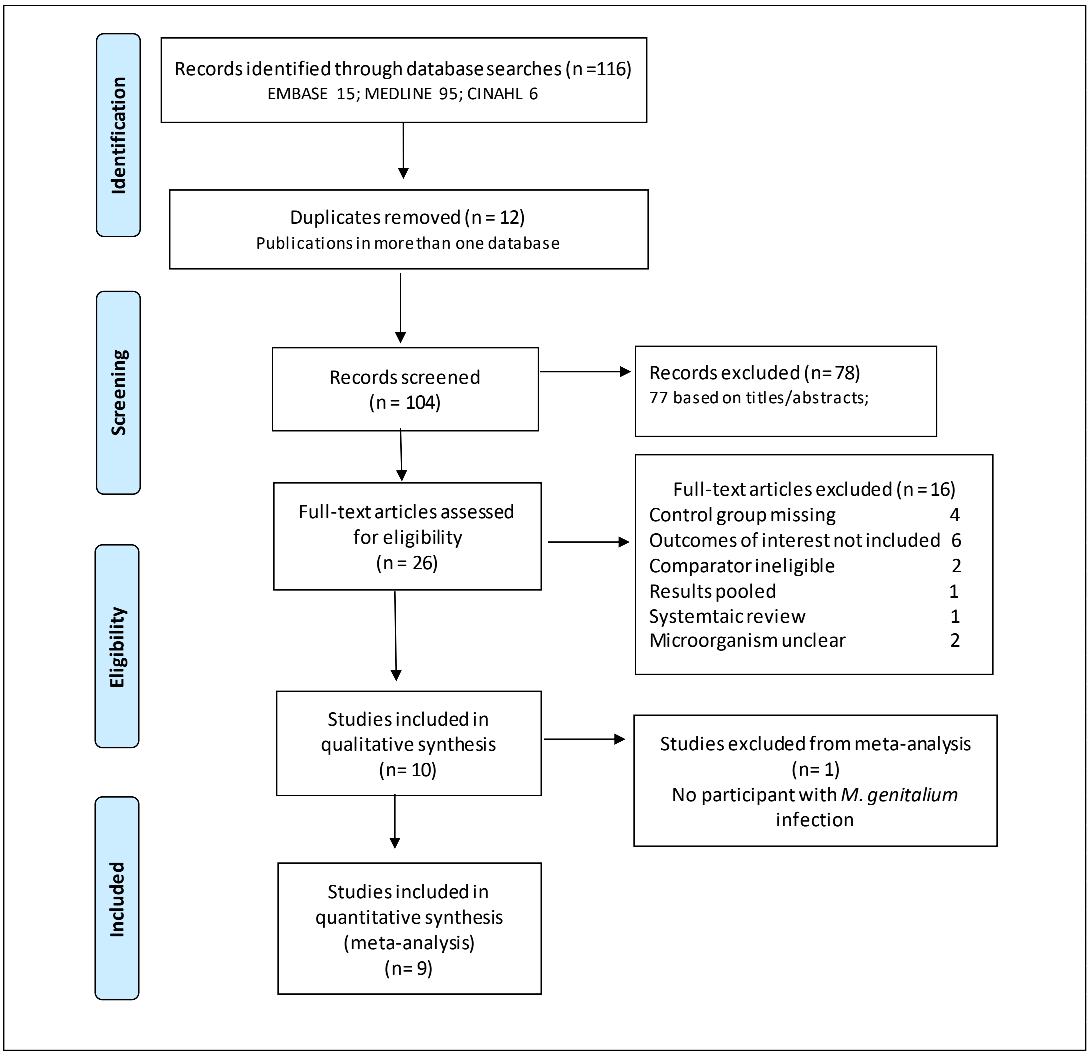

Online supplemental material

**Figure S2: Forest plot of unadjusted effect sizes for association between *M. genitalium* during pregnancy and perinatal death**

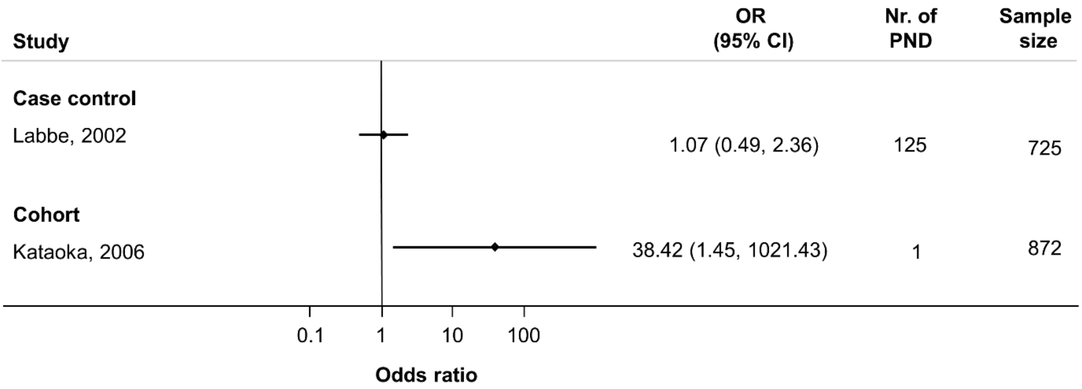


$I^2 = 77\%$

Abbreviations: CI, confidence interval; OR, odds ratio; PND, perinatal death

Notes: For Kataoka et al., there were no *M. genitalium*-infected women who experienced perinatal death. The odds ratio is calculated by adding 0.5 to each cell in the 2x2 table. The sample size is the number of women, excluding the continuity correction.



Frenzer C, *et al. Sex Transm Infect* 2022; 98:222–227. doi: 10.1136/sextrans-2021-055352

Online supplemental material

**Codebook S1, study and population characteristics, appended**

**Codebook S2, Mycoplasma genitalium variables, appended**



Frenzer C, *et al. Sex Transm Infect* 2022; 98:222–227. doi: 10.1136/sextrans-2021-055352
